# A multicenter analysis on the changes of sIgE in China during the early period of COVID‐19 pandemic

**DOI:** 10.1002/iid3.1072

**Published:** 2023-11-07

**Authors:** Yunzhu Li, Linfeng Li

**Affiliations:** ^1^ Department of Dermatology, Beijing Friendship Hospital Capital Medical University Beijing China

**Keywords:** allergen‐specific IgE (sIgE), allergy, China, COVID‐19 pandemic, multicenter analysis

## Abstract

**Objective:**

This study aims to analyze the changes in allergen composition ratio during the early stages of the COVID‐19 outbreak in China and analyze the underlying factors contributing to these alterations.

**Methods:**

A multicenter study approach was employed. A total of 618 male and female patients (0–89 years old) were recruited from the dermatology, pediatrics, and allergy departments of 17 hospitals across 15 Chinese cities between January 2020 and June 2021. Serum samples were collected and subjected to allergen‐specific immunoglobulin E (sIgE) detection using the reversed enzyme allegro‐sorbent test. The allergens included *Dermatophagoides pteronyssinus (D. pteronyssinus)*, *Dermatophagoides farina (D. farina)*, *Ambrosia artemisiifolia (A. artemisiifolia)*, *Salix babylonica (S. babylonica)*, dog dander, alternaria, cockroach, *Artemisia argyi (A. argyi)*, cat dander, house dust, milk, hen's egg, mutton, cod, peanut, beef, soybean, shrimp, crab, and wheat. Each participant was required to exhibit at least one positive sIgE detection result (≥0.35 IU/mL). The chi‐square test was used to analyze the differences between groups.

**Results:**

The positive sIgE proportion of inhalation allergens were significantly lower than that of ingestion allergens. As restrictions on outdoor activities and seafood imports persisted, the positive sIgE proportion of wheat and shrimp exhibited a significant decrease in the spring of 2021 compared to the corresponding period in 2020. Conversely, there was a substantial increase in the positive proportion of crab. The fluctuation in the ratio of *D. pteronyssinus* and *D. farina* appeared to be influenced more by seasonal factors rather than the COVID‐19 pandemic. However, no noteworthy disparities were observed in the proportions of other allergens.

**Conclusion:**

The alterations in allergen composition during the initial phase of the epidemic may be attributed to several factors, such as decreased travel, increased mask usage, reduced carbohydrate consumption, and changes in seafood consumption. However, factors such as season, cultural practices, and customs may also influence the composition of allergens.

## Graphical abstract

Composition ratio of 20 types of specific allergens in China changed under the COVID‐19 pandemic. The special historical background of the epidemic have a certain influence on the change of the proportion of allergens, but it is not the only factor.

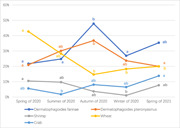

## 1 INTRODUCTION

Allergens are substances that cause allergic reactions in the body. According to the way into the human body, allergens can be divided into ingestion allergens, inhalation allergens, contact allergens, and injection allergens. Among them, ingestion allergens include animal and plant protein, seafood, nuts, and so on. Inhaled allergens include pollen, dust mites, animal epithelial materials, and so on.^1^ According to the different sources, inhalation allergens are divided into indoor allergens and outdoor allergens. Allergens are capable of inducing immunoglobulin E (IgE) production. Allergen‐specific IgE (sIgE) detection is helpful to understand the causes and sensitization status of patients with allergic diseases.^2^

The COVID‐19 outbreak began in Wuhan, China, in December 2019.^3^ On January 20, 2020, China implemented measures for the prevention and control of Class A infectious diseases on it. It was not until 2023 that China lowered the level of control over the COVID‐19 infection.^4^ During this period, residents' outdoor activities and trips were significantly less than in previous years.^5^ With the frequent occurrence of COVID‐19 positive tests on imported seafood, the import of seafood was restricted, and residents' fear of it might also affect the consumption.^6^ As a result, people's exposure to some allergens such as outdoor allergens and people's consumption of seafood was reduced, which might affect people's chances of developing allergies to such allergens. To understand the changes and effect factors in the composition of specific allergens in the early stage of the epidemic, this study collected the sIgE test results of 20 allergens in 17 medical institutions in China from January 2020 to June 2021, and analyzed the differences of these data.

## 2 MATERIALS AND METHODS

### 2.1 Study subjects

The study was not a random sample research. It included all patients who came to the participating departments of 17 hospitals and were willing to participate in the study from January 2020 and June 2021. Screening criteria were not limited to diagnosis, comorbidities, season, region, age, sex, and so on, but patients must have at least one sIgE detection result positive. At last, data of 618 patients with positive results of sIgE detection were collected. They were from dermatology, pediatrics, or allergy departments of 17 hospitals of 15 cities in different regions of China (Table 1). Most of these patients were diagnosed with eczema/atopic dermatitis, urticaria, and/or allergic rhinitis. Among them, 282 were males and 336 were females, ranging in age from 0 to 89, with a median age of 20. The seasons for medical treatment are divided into spring (March to May), summer (June to August), autumn (September to November), and winter (December to February of the following year).

**Table 1:** Distribution of 17 hospitals that participated in the study.

Hospital	City	Number
Peking University Third Hospital	Beijing	11
Beijing Luhe Hospital, Capital Medical University	Beijing	76
Beijing Friendship Hospital, Capital Medical University	Beijing	22
First Affiliated Hospital of Hebei North University	Zhangjiakou	2
Huabei Petroleum Administration Bureau General Hospital	Renqiu	43
People's Hospital of Ningjinxian	Dezhou	9
Shenzhen People's Hospital	Shenzhen	18
The First Affiliated Hospital of Soochow University	Suzhou	46
Tengzhou Central People's Hospital	Tengzhou	90
Tianjin Children's Hospital	Tianjin	48
Tieling Women and Infants Hospital	Tieling	7
The First Affiliated Hospital of Xi'an Jiaotong University	Xi'an	82
Yinan County People's Hospital	Linyi	24
People's Hospital of Yuxi City	Yuxi	46
Zaozhuang Municipal Hospital	Zaozhuang	7
Datong Third People's Hospital	Datong	50
Baoji Central Hospital	Baoji	37

### 2.2 sIgE antibody detection

We collected serum samples from patients during their visit to hospitals. The HOB® Quantitative Allergen‐Specific IgE Antibody REAST Kit (HOB Biotech Group Corp., Ltd.) was used to quantitatively detect serum‐specific IgE levels of patients with method of reversed enzyme allegro‐sorbent test (REAST). The operation procedure is carried out according to the product manual. It briefly consists in the following steps: capture of IgE with a specific antibody absorbed on microtiter wells, incubation with biotinylated liquid allergens, with streptavidin‐peroxidase and chromogenic‐substrate, reading of optical density and interpolation on a reference curve prepared with WHO 75/502 international standard for IgE. Serum sIgE was detected as positive for ≥.35 IU/mL. The 20 items of sIgE detection includes *Dermatophagoides pteronyssinus (D. pteronyssinus)*, *Dermatophagoides farina (D. farina)*, *Ambrosia artemisiifolia (A. artemisiifolia)*, *Salix babylonica (S. babylonica)*, dander of dog, alternaria, cockroach, *Artemisia argyi*, dander of cat, house dust, milk, hen's egg, mutton, cod, peanut, beef, soybean, shrimp, crab, and wheat.

### 2.3 Statistical analysis

SPSS version 22.0 software (SPSS Inc.) was used for data analysis. Positive proportion (%) is the percentage of the positive serum detection to various allergens. The exploration function of SPSS software was used to calculate the 95% confidence interval (CI) of age. The chi‐square test was used to compare the positive proportion of allergens between different groups or time periods. When *n* ≥ 40, T (theoretical frequency) ≥5, Pearson Chi‐square test was used. When *n* ≥ 40, if 1 ≤ T ≤ 5 appears, Yates's correction for continuity was used. When *n* < 40, or T < 1, Fisher's test is used. A two‐tailed *p* < .05 is considered to be statistically significant. The Bonferroni method was used to correct *p* value for multiple comparisons.

## 3 RESULTS

### 3.1 The sIgE positive proportion of the 618 patients

**Figure 1:**
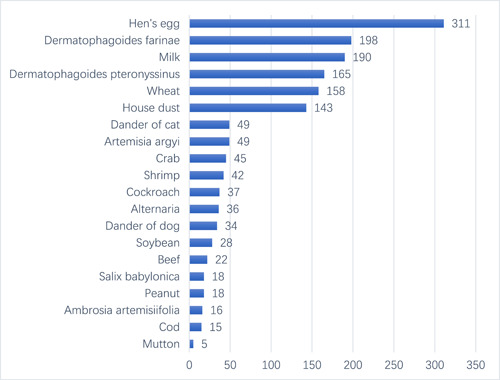
Comparison of the results of 618 patients tested positive for allergen‐specific immunoglobulin E detection from multicenters in China. Hen's egg, *Dermatophagoides* *farinae*, milk, *Dermatophagoides pteronyssinus*, wheat, house dust were the most common allergens of the 618 patients in China from January 2020 to June 2021 under the COVID‐19 pandemic.

Among the 618 patients in this study, patients positive for egg (311/618, 50.32%) were the most common. The remaining items were *D. farina* (198/618, 32.04%), milk (190/618, 30.74%), *D. pteronyssinus* (165/618, 26.70%), wheat (158/618, 25.57%), house dust (143/618, 23.14%), dander of cat (49/618, 7.93%), *A, argyi* (49/618, 7.93%), crab (45/618, 7.28%), shrimp (42/618, 6.80%), cockroach (37/618, 5.99%), alternaria (36/618, 5.83%), dander of dog (34/618, 5.50%), soybean (28/618, 4.53%), beef (22/618, 3.56%), *S. babylonica* (18/618, 2.91%), peanut (18/618, 2.91%), *A. artemisiifolia* (16/618, 2.59%), cod (15/618, 2.43%), mutton (5/618, 0.81%) (Figure 1).

### 3.2 Analysis of age

The age of people who tested positive for inhaled allergens included the following ranges: alternaria: 13.06 years old (95% CI, 8.87–17.26 years old), house dust: 19.65 years old (95% CI, 16.79–22.52 years old), dander of cat: 20.29 years old (95% CI, 15.68–24.90 years old), dander of dog: 23.68 years old (95% CI, 17.28–30.07 years old), *A. artemisiifolia*: 25.70 years old (95% CI, 13.53–37.88 years old), *D. pteronyssinus*: 25.83 years old (95% CI, 23.16–28.50 years old), *S. babylonica*: 25.89 years old (95% CI, 16.58–35.21 years old), *D. farina*: 26.29 years old (95% CI, 23.97–28.62 years old), cockroach: 30.32 years old (95% CI, 25.37–35.27 years old), *A. argyi*: 32.21 years old (95% CI, 27.15–37.28 years old). The age of people who tested positive for ingested allergens included the following ranges: milk: 11.83 years old (95% CI, 9.81–13.85 years old), egg: 15.43 years old (95% CI, 13.51–17.36 years old), peanut: 16.57 years old 95% CI, 6.05–27.08 years old), soybean: 18.79 years old (95% CI, 12.47–25.10 years old), cod: 19.47 years old (95% CI, 7.92–31.02 years old), wheat: 20.32 years old (95% CI, 16.97–23.68 years old), beef: 22.21 years old (95% CI, 13.92–30.50 years old), crab: 25.15 years old (95% CI, 19.95–30.36 years old), shrimp: 29.11 years old (95% CI, 23.25–34.97 years old), mutton: 45.80 years old (95% CI, 27.63–63.97 years old).

### 3.3 Analysis of differences between inhaled and ingested allergens

In this study, a total of 341 patients (341/618, 55.18%) had positive results of inhaled allergen detection, while 494 patients (494/618, 79.94%) had positive results of ingested allergen detection. The former was lower than the latter (*p* < .05). There was no significant difference between male and female in inhaled and ingested allergens (*p* > .05). A total of 308 patients (308/618, 49.84%) tested positive for indoor allergens including *D. farinae*, *D. pteronyssinus*, dander of cat and dog, cockroach, and house dust. While 94 patients (94/618, 15.21%) were positive for outdoor allergens including *S. babylonica*, *A. artemisiifolia*, *A. argyi*, and alternaria. The positive proportions of *S. babylonica* (18/618, 2.91%), *A. artemisiifolia* (16/618, 2.59%), and *A. argyi* (49/618, 7.93%) were lower than that of *D. pteronyssinus* (165/618, 26.70%), *D. farina* (198/618, 32.04%), and house dust (143/618, 23.14%), and there was statistical difference between these groups (*p* < .05).

### 3.4 Change of the positive proportions of various sIgE in the first half of 2020, the second half of 2020, and the first half of 2021

**Figure 2:**
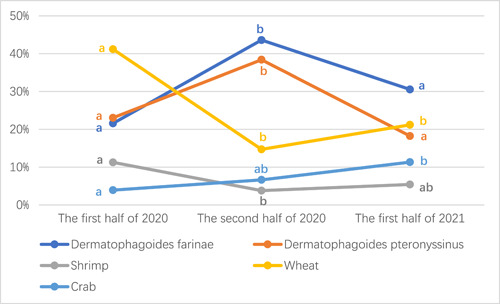
Changes of positive allergen‐specific immunoglobulin E proportion of *Dermatophagoides farina*, *Dermatophagoides pteronyssinus*, wheat, shrimp, and crab in the first half of 2020, the second half of 2021, and the first half of 2021. The positive proportion of *D. pteronyssinus* and *D. farina* increased in the second half of 2020 and decreased in the first half of 2021. The positive proportion of wheat decreased in the second half of 2020 and the first half of 2021. Shrimp decreased significantly in the second half of 2020. Crabs showed an obvious upward trend (a, b, ab: If the same letter was present, there is no difference between the proportions. If the letters are different, there is a significant difference between the proportions. Chi‐square test, a two‐tailed *p* < .05 is considered to be statistically significant).

There were 204 patients positive for sIgE detection in the first half of 2020, and 211 patients in the second half of 2020. While 203 patients were positive in the first half of 2021. Among them, the results of the positive allergens in these three periods showed that egg allergy always ranked the first place, and mutton always ranked the last, but ratio of other allergens had some changes. Statistical analysis showed that the positive proportion of *D. pteronyssinus* and *D. farina* in the second half of 2020 was higher than that in the first half of 2020 (*p* < .05), while the positive proportion in the first half of 2021 was decreased, with a statistical difference compared with the second half of 2020 (*p* < .05). Both *D. pteronyssinus* and *D. farina* had no statistical difference between in the first half of 2020 and in the first half of 2021 (*p* > .05). The positive proportion of wheat in the second half of 2020 and the first half of 2021 was lower than that in the first half of 2020 (*p* < .05), while there was no statistical difference between the second half of 2020 and the first half of 2021 (*p* > .05). There were statistical differences among the first half of 2020, the second half of 2020 and the first half of 2021 in shrimp and crab (*p* < .05). Compared with the first half of 2020, shrimp decreased significantly in the second half of 2020, and rose slightly in the first half of 2021, with statistical differences among the three time periods (*p* < .05). However, the positive proportion of crabs showed an obvious upward trend in the three time periods (*p* < .05). Other items such as dander of cat and alternaria showed a gradual upward trend, but there was no statistical difference (*p* > .05). Both cod and *S. babylonica* had a gradual decline trend, but there was no statistical difference either (*p* > .05). There were no significant differences in egg, milk, peanut, soybean, beef, mutton, dander of dog, cockroach, house dust, *A. artemisiifolia* and *A. argyi* between the first half of 2020, the second half of 2020 and the first half of 2021 (*p* > .05) (Table 2, Figure 2).

**Table 2:** Changes of positive sIgE proportion from 2020 to 2021.

Sort	The first half of 2020	Number	Ratio (N/204) (%)	The second half of 2020	Number	Ratio (N/211) (%)	The first half of 2021	Number	Ratio (N/203) (%)
**1**	Hen's egg	100	49.02	Hen's egg	107	50.71	Hen's egg	104	51.23
**2**	Wheat	84^a^	41.18	*Dermatophagoides farinae*	92^b^	43.60	Milk	73	35.96
**3**	Milk	52	25.49	*Dermatophagoides pteronyssinus*	81^b^	38.39	*D. farinae*	62^a^	30.54
**4**	*D. pteronyssinus*	47^a^	23.04	Milk	65	30.81	House dust	45	22.17
**5**	House dust	45	22.06	House dust	53	25.12	Wheat	43^b^	21.18
**6**	*D. farinae*	44^a^	21.57	Wheat	31^b^	14.69	*D. pteronyssinus*	37^a^	18.23
**7**	Shrimp	23^a^	11.27	*Artemisia argyi*	22	10.43	Crab	23^b^	11.33
**8**	*A. argyi*	15	7.35	Cockroach	19	9.00	Dander of cat	22	10.84
**9**	Dander of dog	13	6.37	Dander of cat	15	7.11	Alternaria	16	7.88
**10**	Dander of cat	12	5.88	Crab	14^ab^	6.64	Dander of dog	14	6.90
**11**	Cockroach	11	5.39	Alternaria	13	6.16	*A. argyi*	12	5.91
**12**	Soybean	10	4.90	Soybean	10	4.74	Shrimp	11^ab^	5.42
**13**	*Salix babylonica*	8	3.92	Shrimp	8^b^	3.79	Soybean	8	3.94
**14**	Cod	8	3.92	*S. babylonica*	7	3.32	Beef	8	3.94
**15**	Crab	8^a^	3.92	Dander of dog	7	3.32	Peanut	7	3.45
**16**	Beef	8	3.92	*Ambrosia artemisiifolia*	7	3.32	Cockroach	7	3.45
**17**	Alternaria	7	3.43	Beef	6	2.84	*A. artemisiifolia*	5	2.46
**18**	Peanut	6	2.94	Cod	5	2.37	*S. babylonica*	3	1.48
**19**	*A. artemisiifolia*	4	1.96	Peanut	5	2.37	Cod	2	0.99
**20**	Mutton	1	0.49	Mutton	3	1.42	Mutton	1	0.49

*Note*: ^a, b, ab^If the letters are different, there is a significant difference between the proportions. Other items without letters indicated no statistical difference (Chi‐square test, a two‐tailed *p* < .05 is considered to be statistically significant).

Abbreviation: sIgE, allergen‐specific immunoglobulin E.

### 3.5 Seasonal variation of positive sIgE proportion of *D. farina*, *D. pteronyssinus*, shrimp, wheat, and crab

**Figure 3:**
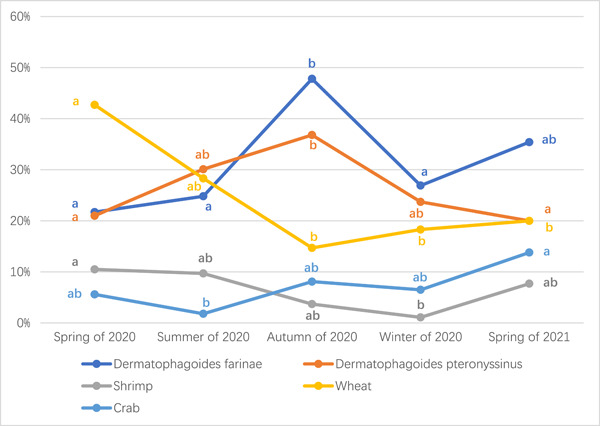
Analysis of seasonal variation of positive allergen‐specific immunoglobulin E proportion of *Dermatophagoides farina*, *Dermatophagoides pteronyssinus*, shrimp, wheat, and crab. The positive proportions of sIgE of *D. pteronyssinus* and *D. farina* were higher in autumn than in other seasons. Shrimp and wheat both showed a downward trend in 2020, and picked up slightly in the spring of 2021. Crab showed an upward trend after the summer of 2020 (a, b, ab: If the same letter was present, there is no difference between the proportions. If the letters are different, there is a significant difference between the proportions. Chi‐square test, a two‐tailed *p* < .05 is considered to be statistically significant).

There were seasonal differences in the positive proportions of sIgE of *D. pteronyssinus* and *D. farina*. In 2020, *D. farinas* was higher in autumn than in other seasons (*p* < .05), but there was no difference between spring, summer, and winter (*p* > .05). The positive proportion in spring of 2021 was higher than in spring of 2020 (*p* < .05). That of *D. pteronyssinus* increased gradually in spring, summer and autumn of 2020, with statistical difference among these three seasons (*p* < .05), and decreased again in winter (*p* > .05). There was no difference between spring of 2020 and spring of 2021 (*p* > .05). Shrimp and wheat both showed a downward trend in 2020, and picked up slightly in the spring of 2021, but were still lower than the same period in the spring of 2020 (*p* < .05). The positive proportion of crab in summer of 2020 was lower than that of the other three seasons, and the autumn was the highest, while the spring of 2021 was significantly higher than spring and other seasons of 2020 (*p* < .05) (Table 3, Figure 3).

**Table 3:** Changes of sIgE of *Dermatophagoides farina*, *Dermatophagoides pteronyssinus*, shrimp, wheat, and crab.

	Spring of 2020	Summer of 2020	Autumn of 2020	Winter of 2020	Spring of 2021
*D. farinae*					
+	31^a^	28^a^	65^b^	25^a^	46^ab^
−	112^a^	85^a^	71^b^	68^a^	84^ab^
Ratio (+/Total)	21.70%	24.80%	47.80%	26.90%	35.40%
*D. pteronyssinus*					
+	30^a^	34^ab^	50^b^	22^ab^	26^a^
−	113^a^	79^ab^	86^b^	71^ab^	104^a^
Ratio (+/Total)	21.00%	30.10%	36.80%	23.70%	20.00%
Shrimp					
+	15^a^	11^ab^	5^ab^	1^b^	10^ab^
−	128^a^	102^ab^	131^ab^	92^b^	120^ab^
Ratio (+/Total)	10.50%	9.70%	3.70%	1.10%	7.70%
Wheat					
+	61^a^	32^ab^	20^b^	17^b^	26^b^
−	82^a^	81^ab^	116^b^	76^b^	104^b^
Ratio (+/Total)	42.70%	28.30%	14.70%	18.30%	20.00%
Crab					
+	8^ab^	2^b^	11^ab^	6^ab^	18^a^
−	135^ab^	111^b^	125^ab^	87^ab^	112^a^
Ratio (+/Total)	5.60%	1.80%	8.10%	6.50%	13.80%

*Note*: ^a, b, ab^If the letters are different, there is a significant difference between the proportions. Chi‐square test, a two‐tailed *p* < .05 is considered to be statistically significant.

Abbreviation: sIgE, allergen‐specific immunoglobulin E.

## 4 DISCUSSION

### 4.1 The potential reasons for the significant difference observed in the positive proportion of indoor inhalation allergens compared to ingestion allergens in sIgE

Among the 20 items of allergen, the most common ingested allergens were egg, milk, and wheat, and the mean age of patients allergic to milk and egg was the lowest, which was related to the altered microbiota composition of gastrointestinal tract in infancy and the early exposure of these foods via gastrointestinal tract and inflamed eczematous skin.^7^ Food‐related allergic reactions decrease with age and are associated with tolerance to common allergenic foods.^8^ Later, due to the increase of children's outdoor activities, microbial exposure and some other factors, the positivity of inhaled allergen increased.^9^

An analysis of allergen detection of 930 allergic patients (0–85 years old) in Zhangzhou (a city in Southern China) in 2017,^10^ an analysis of 731 patients (3‐month‐old to 85 years old) in Zhengzhou (a city in the middle area of China) in 2018,^11^ and an analysis of 11,641 patients (1‐month‐old to 98 years old) in Beijing (a city in northern China) from 2013 to 2017^12^ all showed that the positive rate of inhalation allergen was higher than that of ingestion allergen before. In this study, the sIgE‐positive rate of ingestion allergen was significantly higher than that of inhalation allergen. This may be related to the decrease of outdoor activities and the regulations on wearing masks when traveling. Indoor allergens including *D. pteronyssinus*, *D. farinae*, and house dust accounted for the largest proportion of inhaled allergens, while most outdoor allergens such as *S. babylonica* and *A. artemisiifolia* accounted for significantly less than indoor allergens, further suggesting that people's outdoor activities were relatively less. In China, *A. argyi* is not only an outdoor allergen, it is often used as food ingredients and traditional Chinese medicine,^13^, ^14^ which may be the reason why *A. argyi* has the highest proportion among these outdoor allergens.

### 4.2 The positive sIgE proportion fluctuation of *D. pteronyssinus* and *D. farina* was related to seasonal factors

The positive proportion of *D. pteronyssinus* and *D. farina* increased in the second half of 2020 compared with the first half of 2020, while the positive proportion gradually decreased in the first half of 2021. Further analysis of seasonal differences showed that the peak season of *D. pteronyssinus* and *D. farina* allergies was in autumn, but relatively low in other seasons, which may be the reason for the decrease in the proportion of both allergies in the second half of 2020. *D. pteronyssinus* live in various household items, generally concentrated in carpets, sofas, bedding, and so on, and breed in human dander. *D. farinas* prefer to live in the air, which generally breed in dust, feed, and grain dust, in humid climates and humidity conditions >75%.^15^ *D. pteronyssinus* can be prevented by cleaning, disinfecting, and drying your home. During the epidemic period, people lived more at home, but the positive proportion of *D. farinas* increased in the spring of 2021 compared with the spring of 2020, while it's not the same with *D. pteronyssinus*. The reason is still unknown, which may be related to the attention paid to cleaning and disinfecting home during the epidemic period.

### 4.3 The potential reasons for the changes of positive sIgE proportion of shrimp and crab

Shrimp showed a decreasing trend in 2020, especially in the winter of 2020 when the proportion was the lowest. During the epidemic, Positive results of COVID‐19 test frequently appeared from imported frozen food, which restricted the import of frozen food, and people also had a fear of imported or possibly imported frozen food, including shrimp.^16^ These factors have affected shrimp consumption.^6^ The decline in shrimp allergy may be related to the decline in consumption. The proportion of cod also showed a downward trend, but there was no significant difference in statistical analysis which may due to the number of people allergic to cod was too small. In contrast, the proportion of crabs increased gradually during the epidemic. The Chinese have a long history of eating domestic crab,^17^, ^18^ and the decrease in the consumption of imported seafood may have resulted in a compensatory or relative increase in the consumption of domestic crab, which may be one of the reasons for the significant increase in the proportion of crab.^19^

### 4.4 The possible reason for the changes of positive sIgE proportion of wheat

Wheat is one of the main sources of staple food for the Chinese people. Starting from the second half of 2020, the proportion of wheat allergy declined sharply and remained at a low point in the first half of 2021. With less activity, less energy is expended and the need for staple foods may also decrease. Studies on the diet structure of college students in the COVID‐19 era found that more young people's consumption and intake of staple food had decreased,^20^, ^21^ but whether this caused the decrease in wheat allergy rate still needs to be further studied.

### 4.5 Limitations of this study

In addition, there are some limitations on this study. Limited sample size and selection bias exist. The patients included in the study were from 17 hospitals in different regions of China, which may not be representative of the entire population. The study focused on detecting 20 specific allergens, which may not cover the full spectrum of allergens relevant to the population. A long‐term follow‐up data is still needed to provide more insights into the trends and changes in allergen sensitization over an extended period.

## 5 CONCLUSIONS

The data of 618 patients showed that egg, *D. farina*, milk, *D. pteronyssinus*, and wheat were the main allergens in China during the COVID‐19 outbreak from January 2020 to June 2021. The proportion of inhalation allergens was significantly lower than that of ingestion allergens. Most of the inhaled allergens were indoor allergens. In addition, the fluctuation of *D. farina* and *D. pteronyssinus* allergy was also related to seasonal factors. The proportion of wheat and shrimp allergy decreased, while the proportion of crab allergy increased. There was no significant difference in the proportion of other allergens. The special historical background of the epidemic may have a certain influence on the change of the proportion of allergens, such as reduced travel, mask application, reduced demand for carbohydrate intake, and changes in seafood consumption, but there were other factors such as season, manners and customs that affect the composition of allergens.

## AUTHOR CONTRIBUTIONS

Yunzhu Li contributed to the data curation, formal analysis, writing the original draft, review, and editing the article. Linfeng Li contributed to the conceptualization, project administration, resources, software, supervision, validation, and visualization. All authors read and approved the final manuscript. All authors have agreed to be accountable for all aspects of the work.

## CONFLICT OF INTEREST STATEMENT

The authors declare no conflict of interest.

## ETHICS STATEMENT

This study was approved by the Ethical Committee of Beijing Friendship Hospital, Capital Medical University, Beijing, China (approval number: 2019‐P2‐108‐01). Before entering the study, patients provided written informed consent; for all participants aged under 18, written informed consent was obtained from parents/legal guardians.

## Data Availability

Data in this study are available from the corresponding author upon request.
